# KSTRV1: A scene text recognition dataset for central Kurdish in (Arabic-Based) script

**DOI:** 10.1016/j.dib.2025.111648

**Published:** 2025-05-14

**Authors:** Sardar Omar Salih, Karwan Jacksi

**Affiliations:** a Web Technology Dept., Duhok Technical Institute, Duhok Polytechnic University, Duhok, Iraq; b Information Technology Dept., Technical College of Informatics, Akre University of Applied Sciences, Duhok, Iraq; c Semantic Web Lab., University of Zakho, Kurdistan Region of Iraq, Iraq

**Keywords:** Text dataset, Scene text recognition (STR), Kurdish scene text recognition (KSTR), Multilingual text recognition, Synthetic data for OCR, Non-latin script OCR, Natural scene

## Abstract

Scene Text Recognition (STR) has advanced significantly in recent years, yet languages utilizing Arabic-based scripts, such as Kurdish, remain underrepresented in existing datasets. This paper introduces KSTRV1, the first large-scale dataset designed for Kurdish Scene Text Recognition (KSTR), addressing the lack of resources for non-Latin scripts. The dataset comprises 1,420 natural scene images and 19,872 cropped word samples, covering Kurdish (Sorani and Badini dialects), Arabic, and English. Additionally, 20,000 synthetic text instances have been generated to enhance the dataset’s diversity, quantity, and quality by incorporating varied fonts, orientations, distortions, and background complexities.

KSTRV1 captures the multilingual landscape of the Kurdistan Region while addressing real-world challenges like occlusion, lighting variations, and script complexity. The dataset includes detailed annotations with bounding boxes, language identification, and text orientation labels, ensuring comprehensive support for training and evaluating STR models. By providing both natural and synthetic data, KSTRV1 enables the development of robust text recognition models, particularly for Central Kurdish, a low-resource language.

The KSTRV1 dataset is publicly available at https://doi.org/10.5281/zenodo.15038953 and is expected to significantly contribute to research in multilingual STR, document analysis, and optical character recognition (OCR), facilitating more inclusive and accurate text recognition systems.

Specifications Table

**Table:** 

Subject	Computer Sciences
Specific subject area	Scene Text Recognition (STR) for non-Latin script such as Kurdish
Type of data	.jpg: scene Images - real images and synthetical images .txt: text files for annotation
Data collection	The images were captured by volunteers across the Kurdistan Region, specifically in Erbil, Duhok, and Sulaymaniyah, offering a diverse visual representation of the region's landscapes and urban environments. These images were taken under various conditions, including daytime and nighttime settings, and from different distances and angles. The dataset encompasses a broad range of subjects, including markets, advertisements, informational signs, addresses, traffic signals, public events, and other text-rich environments. The text within the images reflects the multilingual nature of the region, containing Central Kurdish (Sorani and Badini dialects), Arabic, and English. The dataset comprises 1,420 natural scene images and 19,872 cropped word samples, covering Kurdish (Sorani and Badini dialects), Arabic, and English. Additionally, 20,000 synthetic text instances have been generated to enhance the dataset’s diversity, quantity, and quality by incorporating varied fonts, orientations, distortions, and background complexities.
Data source location	The images were captured by volunteers across the Kurdistan Region, specifically in Erbil, Duhok, and Sulaymaniyah, offering a diverse visual representation of the Iraqi Kurdistan region's landscapes and urban environments.
Data accessibility	Repository name: KSTRV1 (Kurdish STR) Version 1 at zenodo.org Data identification number: 10.5281/zenodo.15038953 Direct URL to data: https://doi.org/10.5281/zenodo.15038953
Related research article	none

## 1 Value of the Data


 

• KSTRV1, the first large-scale dataset designed for Kurdish Scene Text Recognition (KSTR), addressing the lack of resources for non-Latin scripts.
• KSTRV1 captures the multilingual landscape of the Kurdistan Region- Iraq while addressing real-world challenges like occlusion, lighting variations, and script complexity.
• The dataset includes detailed annotations with bounding boxes, language identification, and text orientation labels, ensuring comprehensive support for training and evaluating STR models. By providing both natural and synthetic data, KSTRV1 enables the development of robust text recognition models, particularly for Central Kurdish, a low-resource language.
• The KSTRV1 dataset is publicly available at https://doi.org/10.5281/zenodo.15038953 and is expected to significantly contribute to research in multilingual STR, document analysis, and optical character recognition (OCR), facilitating more inclusive and accurate text recognition systems.
• A key objective of this dataset is to enhance detection and recognition performance for Central Kurdish, an Arabic-based language with limited computational resources and minimal prior research.
• Text recognition from images presents significant challenges due to various factors. Images are often captured randomly in natural environments, leading to distortions and environmental interferences, such as poor lighting conditions and character occlusions. Additionally, text may appear in non-standard orientations, including rotations and curved arrangements, or in a wide range of stylized formats, from advertisements and artistic calligraphy to distorted or partially obscured characters, all of which further complicate recognition.
• 20,000 synthetic text instances have been generated for these three languages, enhancing the dataset’s quantity and diversity while incorporating natural text features.

## 2 Background

Scene Text Recognition (STR) has gained significant attention in recent years [1], particularly for languages with complex scripts such as Arabic [2,3], Persian [4], and Urdu [5], and other languages that use the Arabic-based script. While extensive datasets exist for the most widely spoken languages [1], datasets specifically designed for Kurdish Scene Text Recognition (KSTR) remain underdeveloped [6]. The closest available resources are datasets focused on Arabic and Persian, whose scripts share similarities with Kurdish but differ in certain characters.

Several notable datasets have been introduced for Arabic scene text recognition, providing valuable insights that can inform research on Kurdish STR due to the shared script characteristics. The EASTR-42K dataset [3] serves as a significant resource, comprising over 42,000 labeled text images extracted from natural scenes. Its large-scale and diverse nature has contributed to improved Arabic text recognition across various environments, making it a relevant reference for Kurdish text recognition research. Similarly, the EvArEST dataset [2] offers an evaluation framework for Arabic scene text recognition by providing a substantial dataset of Arabic text extracted from 510 real-world images, facilitating benchmarking across multiple STR models.

Earlier studies, such as the KHATT dataset [7], were developed for Arabic handwriting text recognition but have also contributed to the broader recognition of texts in the Perso-Arabic script. Although KHATT primarily focuses on handwritten rather than scene text, the challenges posed by character shape variations in Arabic are similar to those encountered in Kurdish. Furthermore, the ICDAR 2017 RRC-Arabic dataset [8] introduced Arabic scene text images for multilingual text recognition tasks, advancing the field by enabling comparisons between STR models for Arabic and other related scripts.

For Persian, Farsi Text Recognition (FATR) [9] Dataset consists of 12935 instance images for training and 3529 instance text images for testing, provides a significant resource for recognizing Persian text from natural scenes. The Perso-Arabic script used in Persian closely resembles the Kurdish script, particularly in the Sorani and Badini dialects. However, Kurdish includes unique characters, such as  which necessitate tailored datasets and specialized models to ensure accurate text recognition.

Although datasets for Arabic, Urdu, and Persian provide valuable insights, a dedicated dataset for Kurdish Scene Text Recognition (KSTR) remains unavailable. To bridge this gap, the KSTRV1 dataset has been introduced as the first dataset specifically developed for Kurdish STR. The KSTRV1 dataset comprises 19,872 labeled text samples extracted from natural scene images, covering both the Sorani and Badini dialects, as well as supplementary samples in Arabic and English. To augment the dataset’s volume and quality, synthetically generated labeled images have been integrated, thereby enhancing its utility for text recognition tasks.

KSTRV1 supports the development of robust STR models by incorporating the unique characters of Kurdish  which are absent in standard Arabic and Persian scripts. This work builds upon existing datasets such as EASTR-42K, EvArEST, KHATT, and PESTD, adapting their methodologies to meet the specific requirements of Kurdish STR. The introduction of KSTRV1 represents a significant advancement, providing the first large-scale resource for Kurdish scene text recognition and establishing a foundation for further research in multilingual STR tasks involving Kurdish. Table 1 provides an overview of various datasets developed for text detection and recognition in Arabic-based scripts.

**Table 1:** Datasets for scene text recognition in Arabic, Persian, and Urdu.

No.	Dataset	Samples	Language	Tasks	Orientation	Refs.	Year
1	ARASTEC	260	Arabic	Detection & Recognition	Horizontal & rotated	[10]	2015
2	KHATT	∼3,000,000	Arabic	Handwriting Recognition	Horizontal	[7]	2016
3	IIIT-ILST	1000	three Indic scripts Devanagari, Telugu and Malayalam.	Detection & Recognition	Horizontal & rotated	[11]	2017
4	ICDAR 2017 RRC-Arabic	∼1,000	Arabic	Multilingual Text Recognition	Horizontal & rotated	[8]	2017
5	EvArEST	510+ images (with multiple text instances)	Arabic	Text Detection & Recognition	Horizontal & rotated	[2]	2018
6	MLT	20,000	Multilanguage with Arabic	Detection & Recognition	Horizontal & rotated	[12]	2019
7	EASTR-42K	42,000+	Arabic	Text Detection & Recognition	Horizontal & rotated	[3]	2019
8	Farsi Scene Text	∼10,000	Persian	Text Detection & Recognition	Horizontal & rotated	[13]	2019
9	Urdu text in natural scene images:	∼500	Urdu	Text Detection Recognition	Horizontal	[14]	2021
10	Handwritten digits and characters	315,000 70,000 digits 245,000 chars	Kurdish	Recognition	Horizontal	[15]	2021
11	EMNIST-like for Handwritten	406000	Kurdish	Recognition	Horizontal	[16]	2024
12	FATR	12935 training 3529 testing	Persian	Recognition	Horizontal & rotated	[9]	2025

## 3 Data Description

**Fig. 1:**
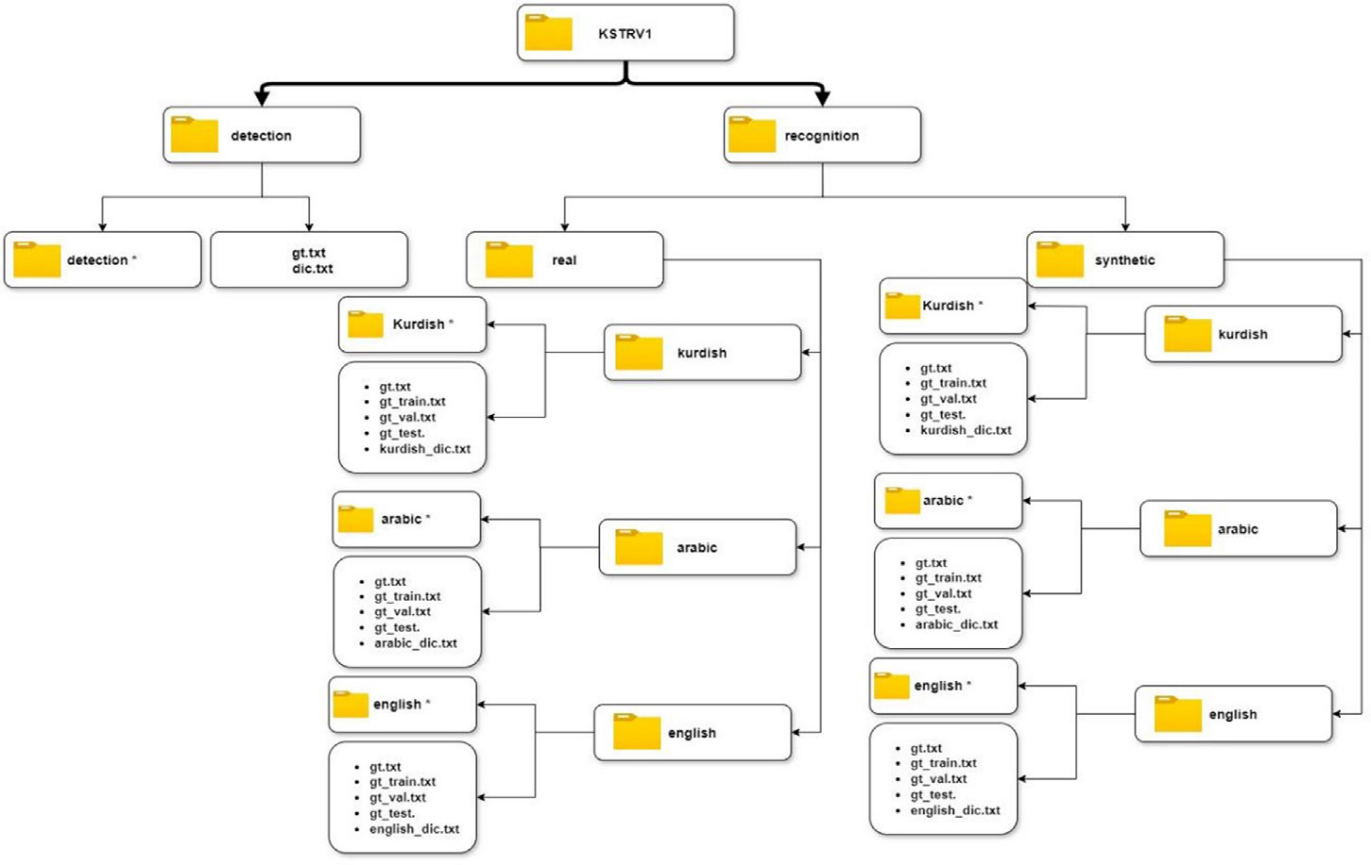
Structure of the KSTRV1 dataset consists of two main folders detection and recognition, each contains images with corresponds ground truth annotations text files.

The dataset consists of two main folders: detection and recognition. The detection folder contains 1,420 images captured from natural scenes across the Kurdistan region, including Duhok, Erbil, and Sulaymaniyah. It also includes two annotation files: gr.txt, which contains instance text annotations for all images, and dic.txt, which lists all unique characters present in the dataset. The recognition folder is divided into two subdirectories: real, which contains real text image instances cropped from natural scene images, and Synthetic, which includes text images generated using various techniques. Both folders are further divided into three subfolders, each representing a different language, Kurdish, Arabic and English in addition to the annotated files for train, validation, test and lists all unique characters. Fig. 1 bellow illustrates the visual structure of dataset, note the folder with * contain images.

**Fig. 2:**
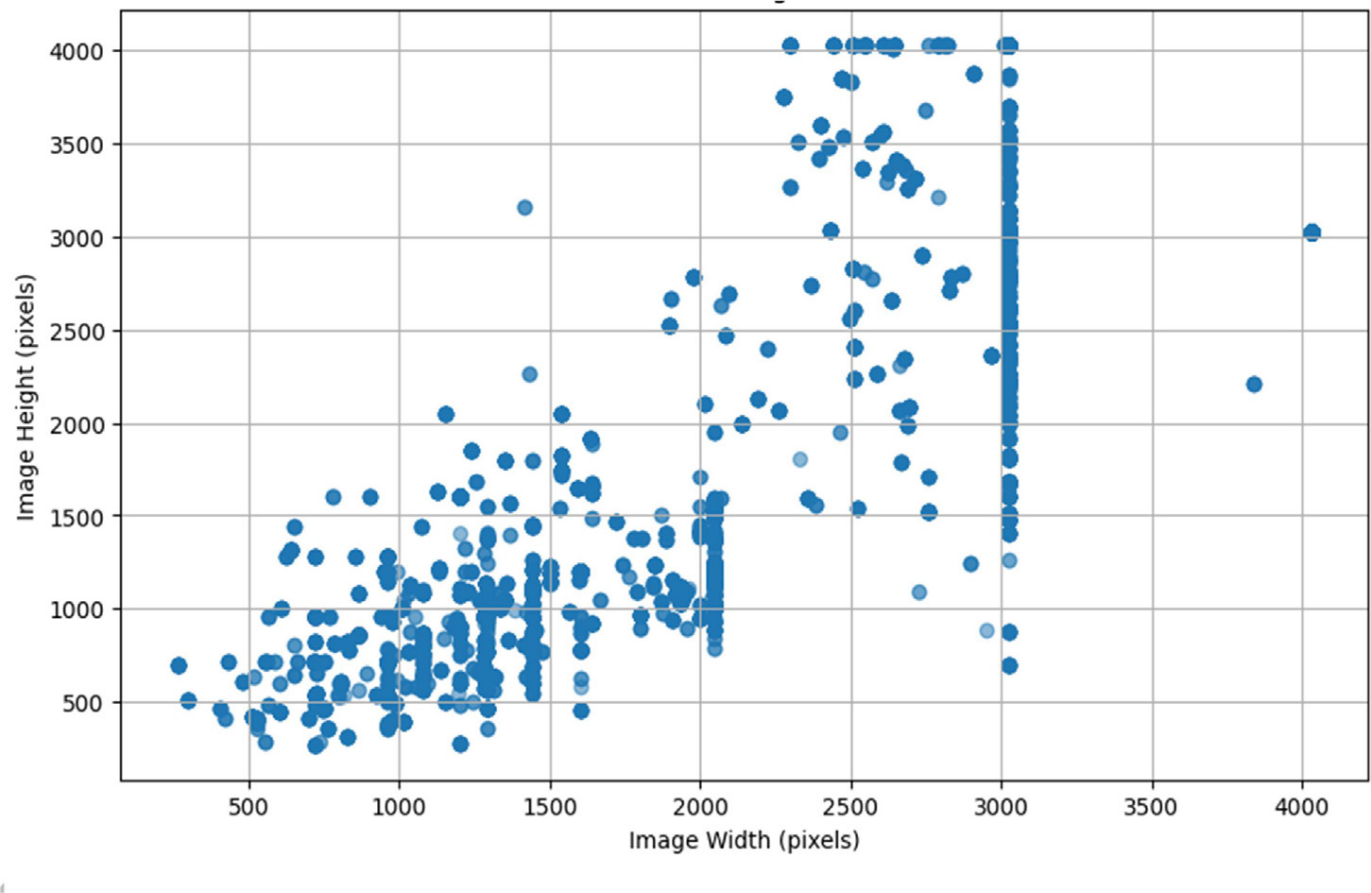
Distribution of image resolutions.

The dataset includes a wide range of image resolutions, with natural scene images captured using various mobile devices. A scatter plot, as shown in Fig. 2, shows the distribution of image widths and heights, with clusters around standard mobile phone resolutions such as 1000 × 1500, 2000 × 3000, and 2500 × 4000 pixels.

**Fig. 3:**
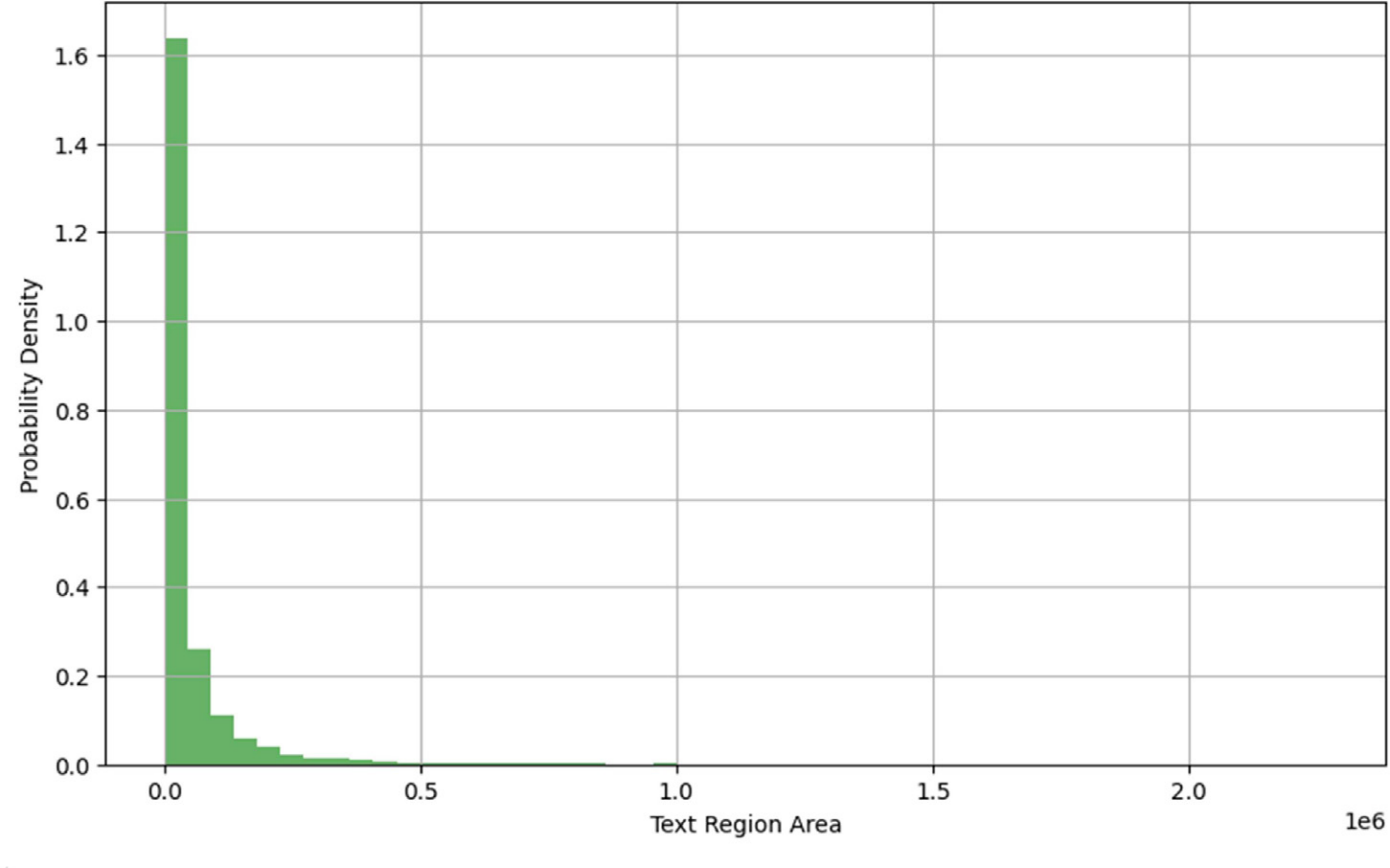
Probability distribution of text region areas.

Additionally, the distribution of text region areas, as shown in Fig. 3, reveals that most text instances occupy relatively small spaces within images. This skewed distribution is typical of natural scenes and reflects the real-world occurrence of text on signs, labels, and posters. The variability in both image sizes and text region scales contributes to the dataset’s utility in training generalizable STR models.

## 4 Experimental Design, Materials and Methods

### 4.1 Kurdish Language, Dialects, and Orthographic Characteristics

The Kurdish language belongs to the Indo-European language family [17] and is spoken across Iraq, Iran, Turkey, and Syria. Despite its widespread use, Kurdish does not have an independent state. Various dialects are spoken, with Sorani and Kurmanji being the most widely used. The Sorani dialect employs an Arabic-based script, whereas Kurmanji utilizes a Latin-based script. The number of native Kurdish speakers is estimated to be between 20 and 30 million [18].

**Fig. 4:**
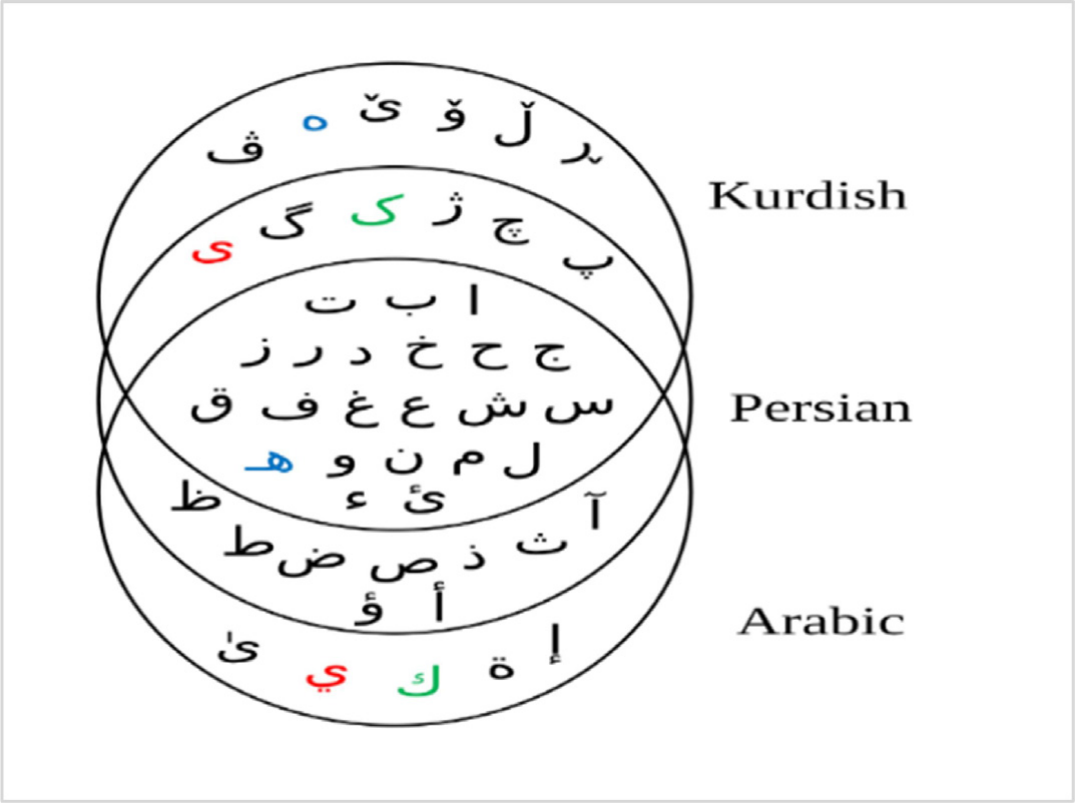
Shared and language specific characters across Arabic, Persian, and Kurdish.

The text in the dataset images is written in Central Kurdish, which includes the Sorani and Badini dialects. Its alphabet consists of characters derived from Arabic, Persian, and additional Kurdish-specific letters, such as [ە, ێ, ۆ, ڵ, ڕ, and ڤ] as illustrated in Fig. 4.

The shared characters among Kurdish, Arabic, and Persian may appear similar; however, the distinct characteristics of the Kurdish script present significant challenges when adapting Arabic or Persian datasets for Central Kurdish text recognition. As illustrated in Table 2, the Central Kurdish alphabet comprises 34 letters, each of which can change its form depending on its position within a word.

**Table 2:** Central Kurdish alphabet.

For example, the Arabic Letter Yeh with Small V (ێ) modifies its shape based on whether it appears in isolation, at the beginning, middle, or end of a word, as demonstrated in Table 3.

**Table 3:** Shape variations of the letter “ێ” based on word position.

The Kurdish letter “ێ” (Arabic Letter Yeh with Small V) is typically transliterated as “ê” in the Latin-based Kurdish alphabet used for Kurmanji. It represents a long /eː/ sound, similar to the “é” in French (as in *café*) or the “e” in “they” in English. The letter in Central Kurdish exhibits shape variations depending on its position within a word, as illustrated in Table 2:

The Central Kurdish script is considered a complex script due to several unique characteristics. It is cursive and follows a right-to-left writing direction, making text processing more challenging. Additionally, ligatured characters and contextual shape variations require recognition models to adapt based on a character’s position within a word. The script includes diacritics, which introduce an additional layer of complexity for text recognition systems. Despite being spoken by a significant population, Central Kurdish is classified as a low-resource language due to the limited availability of datasets and computational tools. Additionally, the calligraphic nature of the script results in variations in writing styles, which must be carefully considered and addressed in text detection and recognition tasks. These factors collectively contribute to the challenges associated with developing robust solutions for Kurdish text recognition [19].

### 4.2 Collection

**Fig. 5:**
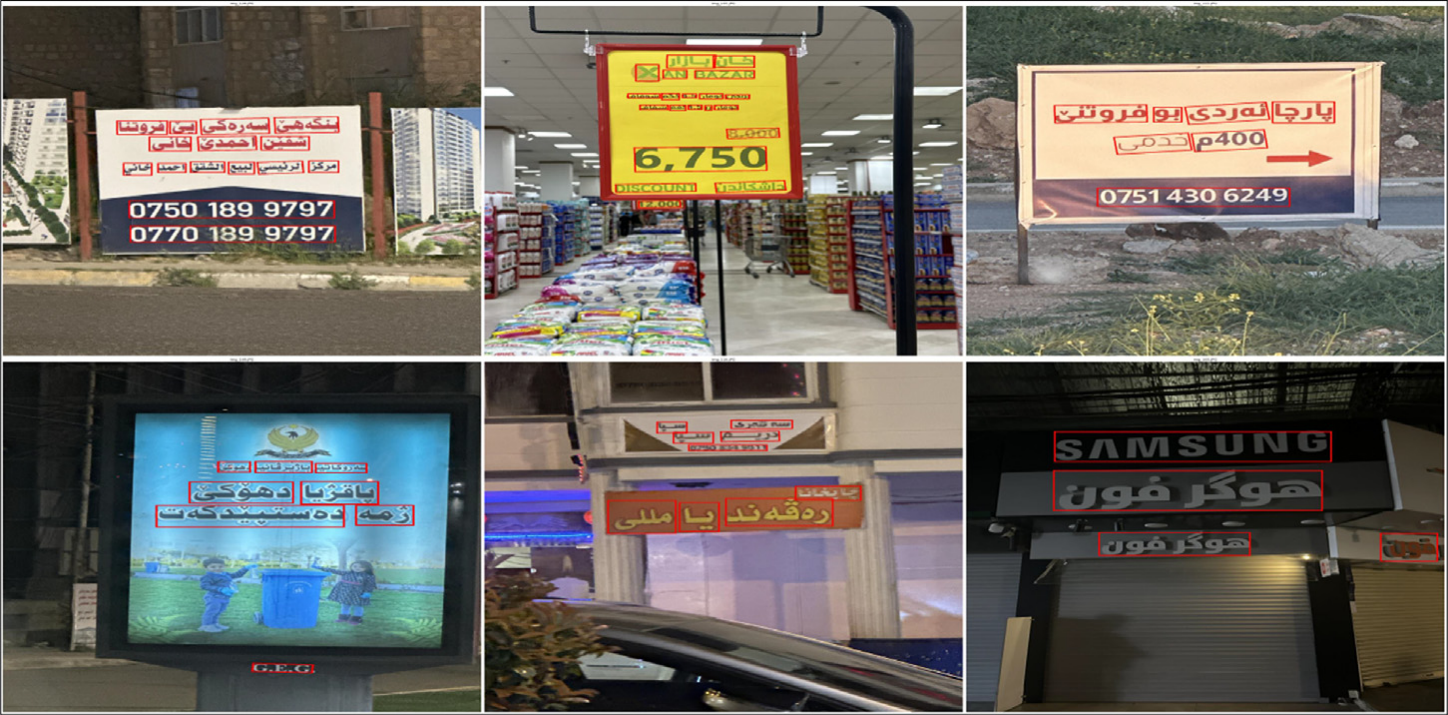
Sample images captured from various locations in the Kurdistan region.

The images were captured by volunteers across the Kurdistan Region, specifically in Erbil, Duhok, and Sulaymaniyah, offering a diverse visual representation of the region's landscapes and urban environments. These images were taken under various conditions, including daytime and nighttime settings, and from different distances and angles. The dataset encompasses a broad range of subjects, including markets, advertisements, informational signs, addresses, traffic signals, public events, and other text-rich environments. The text within the images reflects the multilingual nature of the region, containing Kurdish, Arabic, and English. A few representative samples of these images are provided in Fig. 5.

**Fig. 6:**
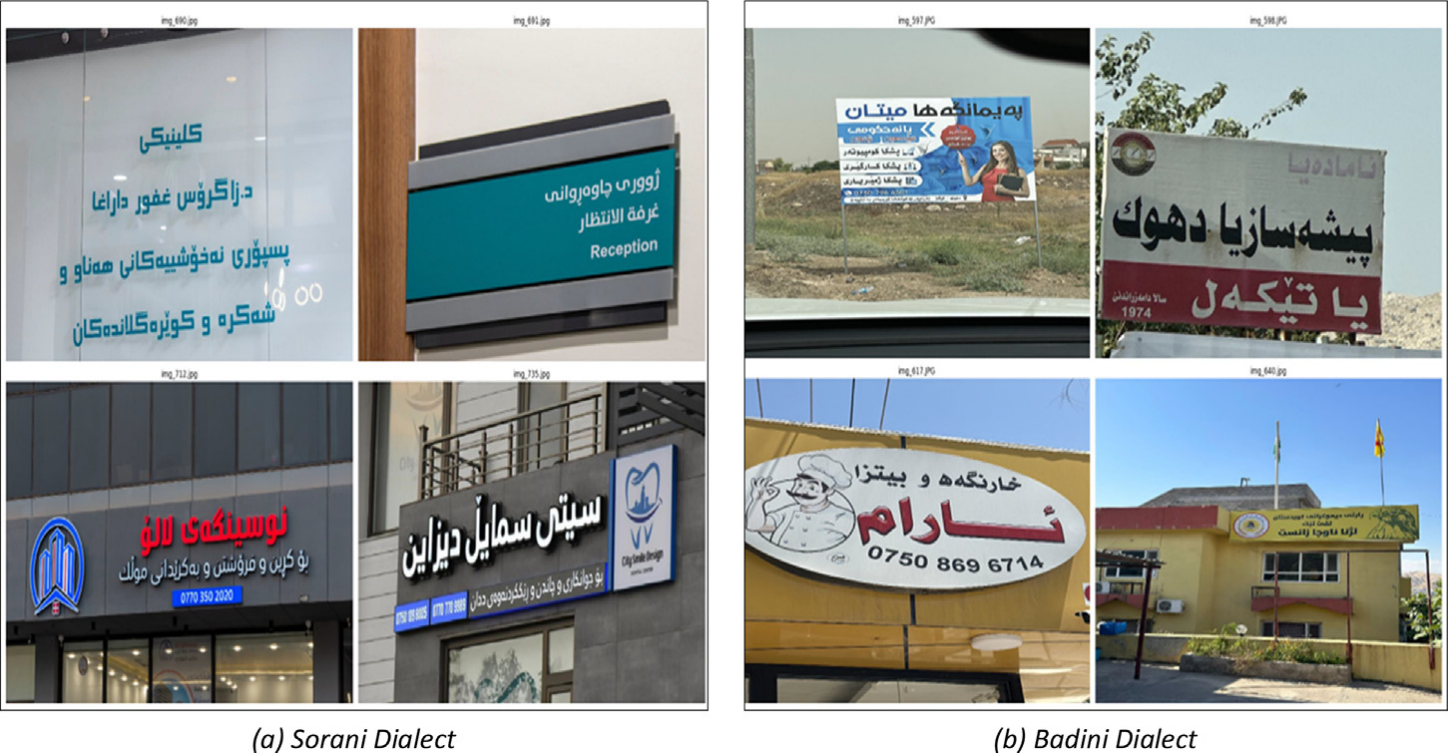
Dataset samples of Kurdish text in sorani and badini dialects.

A detailed analysis of 1,420 images revealed that 409 images contained only Kurdish script, as illustrated in Fig. 6. Notably, no images exclusively containing Arabic or English text were identified. It is important to highlight that both Sorani and Badini, the two primary dialects of Central Kurdish, utilize the Arabic-based script. Sorani is predominantly spoken in Sulaymaniyah and most parts of Erbil, whereas Badini is spoken in Duhok and some parts of Erbil. The dataset includes 785 images representing Badini and 635 images representing Sorani. A selection of sample images showcasing these two dialects is presented in Fig. 6.

### 4.3 Annotation Process

**Fig. 7:**
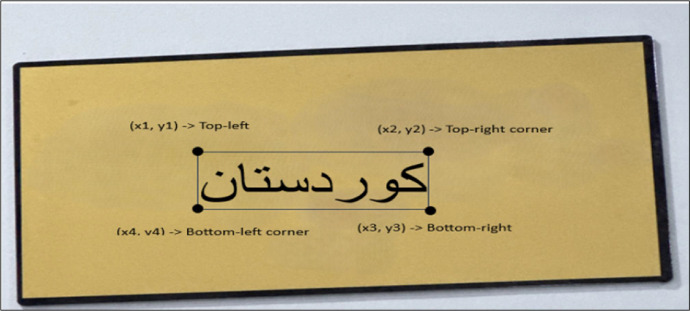
Examples of annotated images with bounding boxes consisted of four points.

The image text annotation process involves accurately identifying each text instance within an image at the word level. This is accomplished by defining a bounding box around each word using a clockwise sequence of four points, forming either a rectangle for regular text annotation and a quadrilateral for irregular text annotation, for this task, the **PPOCRLabel** [20] annotation tool is utilized, which provided an efficient and user-friendly interface for managing multilingual text regions. as illustrated in Fig. 7. Each point's coordinate is determined based on its distance from the left and top edges of the image.

The annotation process also considers the linguistic orientation of the text. Kurdish and Arabic texts are annotated from right to left, aligning with their natural writing direction, whereas English text is annotated from left to right.

**Fig. 8:**
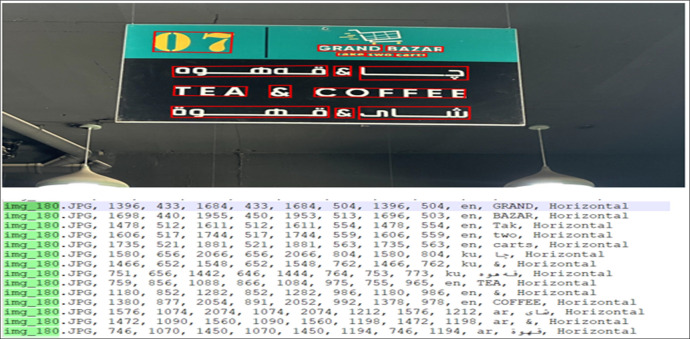
Example of annotation format – image (Top) and corresponding annotation file text-based (Bottom) formatting from left to right, image name, bounding points, language, text in bounding box and text-orientation.

**Fig. 9:**
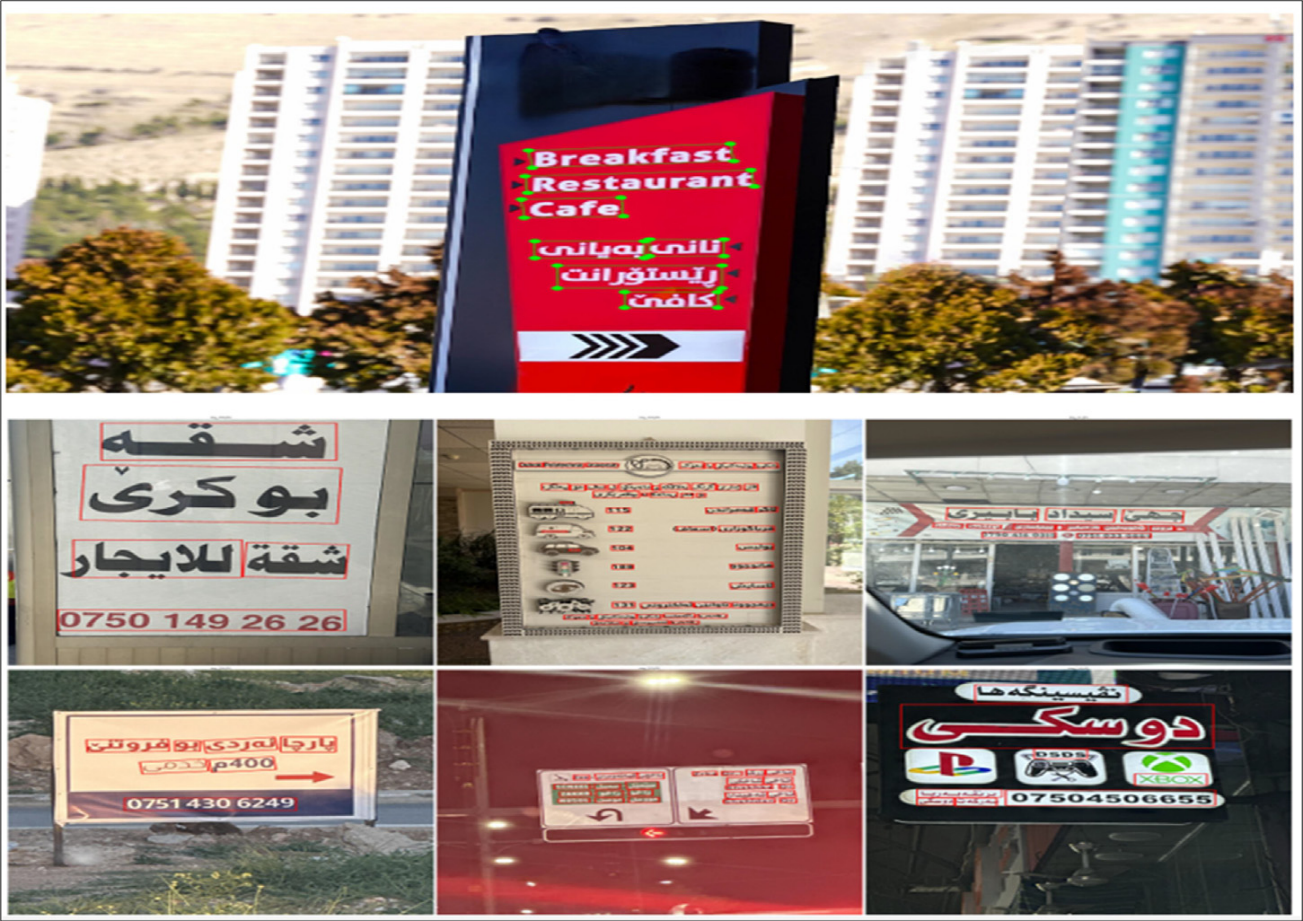
Additional samples of annotated images shows red bounding box around text on images.

The annotated text file is stored in a comma-separated format, beginning with the image name, followed by the set of four points representing the bounding box, and additional metadata. This metadata includes the language of the text, the textual content within the bounding box, and the text orientation (e.g., horizontal, vertical, or skewed). An example of this annotation format is provided in Fig. 8, where the annotated image is shown at the top and its corresponding annotation file is displayed at the bottom. Fig. 9 presents additional examples of annotated images.

As part of the dataset analysis, the area and aspect ratio of each annotated text region were computed using its bounding quadrilateral. This was followed by plotting the probability distributions of text region sizes and image resolutions to characterize the dataset's structural diversity.

### 4.4 Challenges

Text recognition from images presents significant challenges due to various factors. Images are often captured randomly in natural environments, leading to distortions and environmental interferences, such as poor lighting conditions and character occlusions. Additionally, text may appear in non-standard orientations, including rotations and curved arrangements, or in a wide range of stylized formats, from advertisements and artistic calligraphy to distorted or partially obscured characters, all of which further complicate recognition.

Moreover, the unique orthographic features of the Arabic-based Kurdish script, including the use of diacritics and ligatures, add substantial complexity, potentially reducing recognition accuracy. Variability in backgrounds and font styles introduces additional challenges, making it difficult for recognition models to achieve optimal performance.

**Fig. 10:**
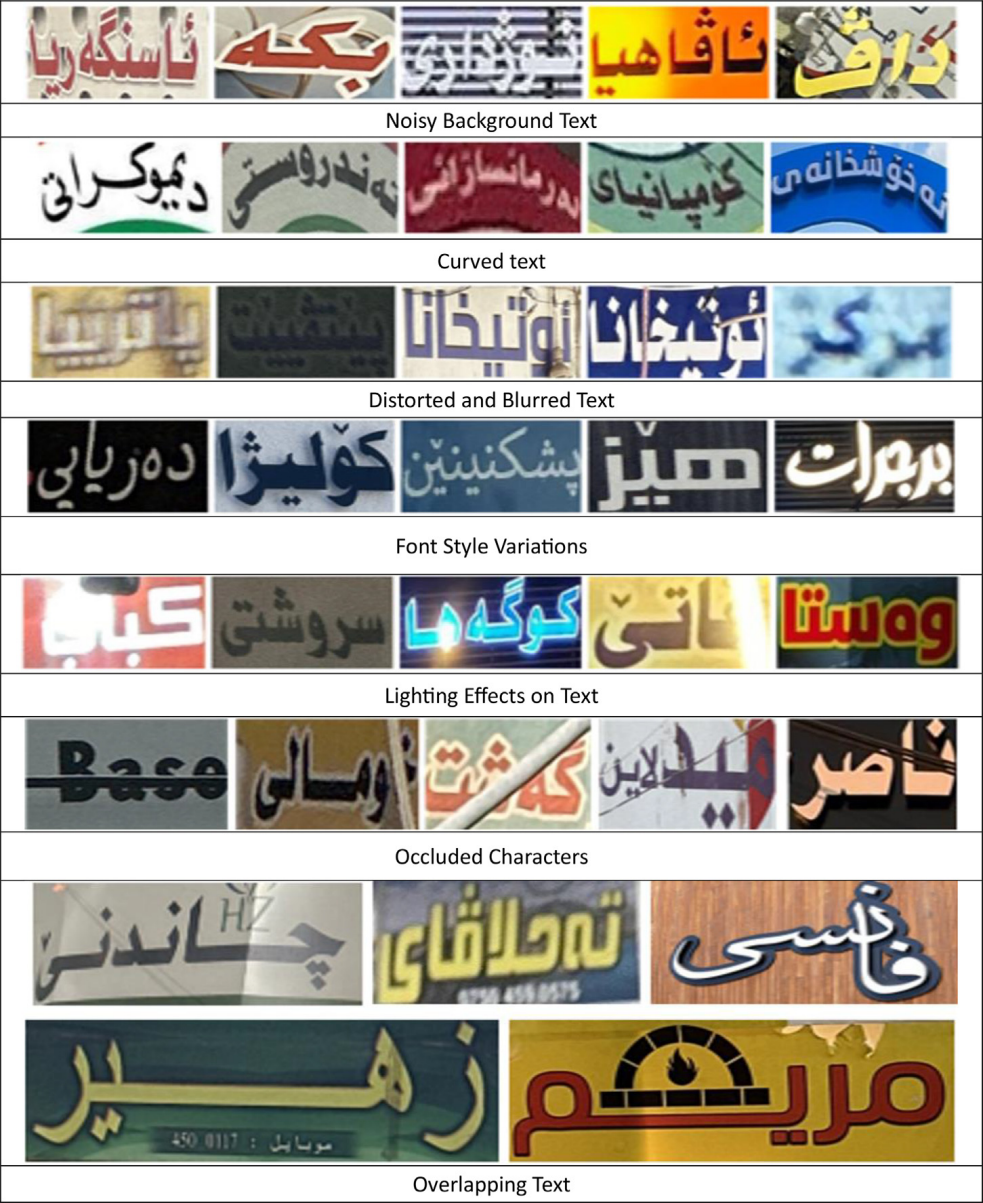
Samples of challenge in cropped images affecting text recognition.

**Fig. 11:**
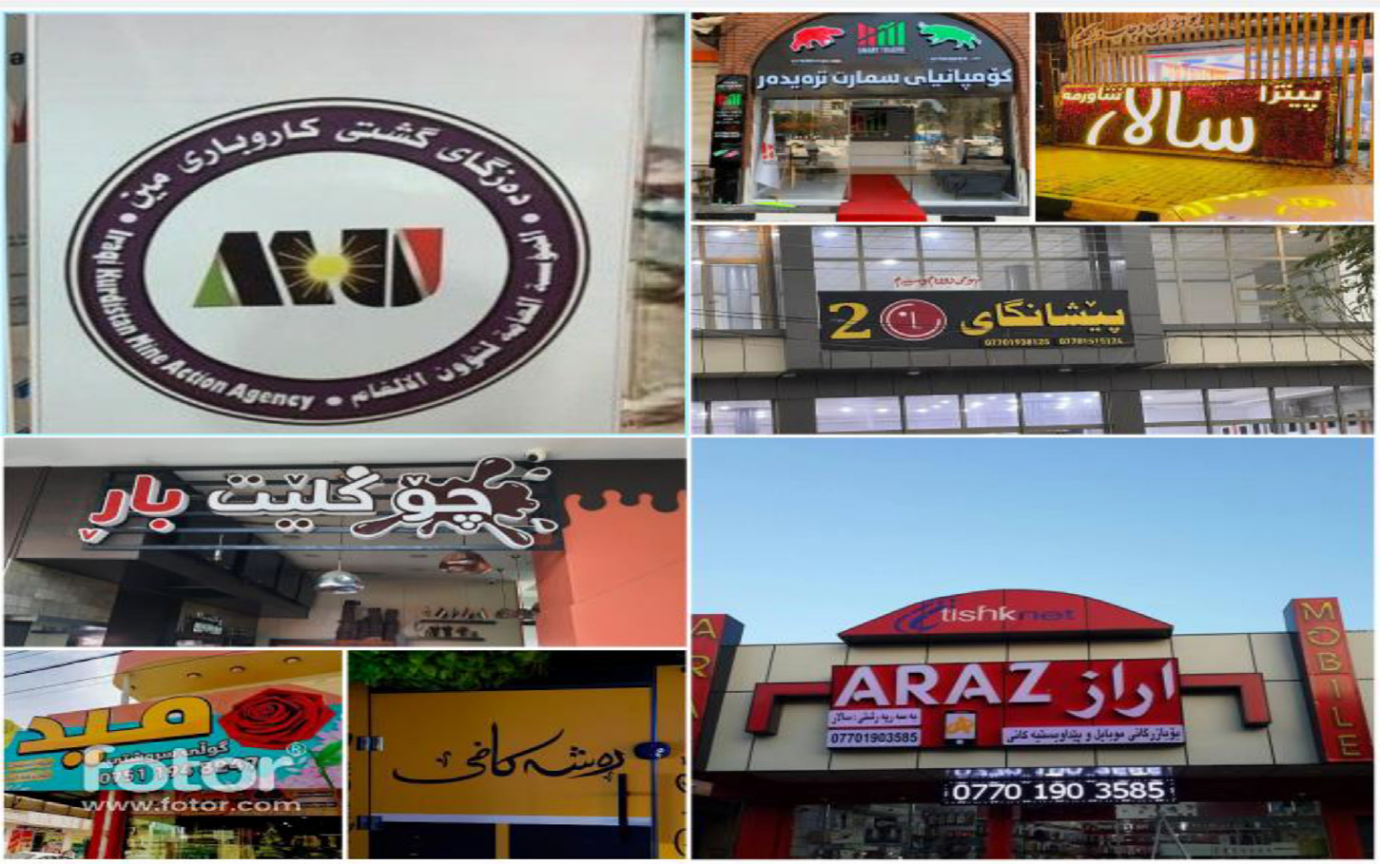
Samples of challenges in text detection from images.

Samples from the dataset illustrate the challenges in both detection and recognition highlighting the difficulties in accurately identifying and extracting text. Fig. 10, Fig. 11 present examples from the dataset, with Fig. 10 showcasing challenges related to image recognition, such as noise, distortions, and lighting effects, while Fig. 11 highlights text detection difficulties in real-world images.

### 4.5 Synthetic Data and Data Augmentation

As previously mentioned, the KSTRV1 dataset includes over 19,872 labelled text samples extracted from natural scene images. These samples represent a mix of three languages (Kurdish, Arabic, and English) and include Kurdish dialects (Sorani and Badini).

**Fig. 12:**
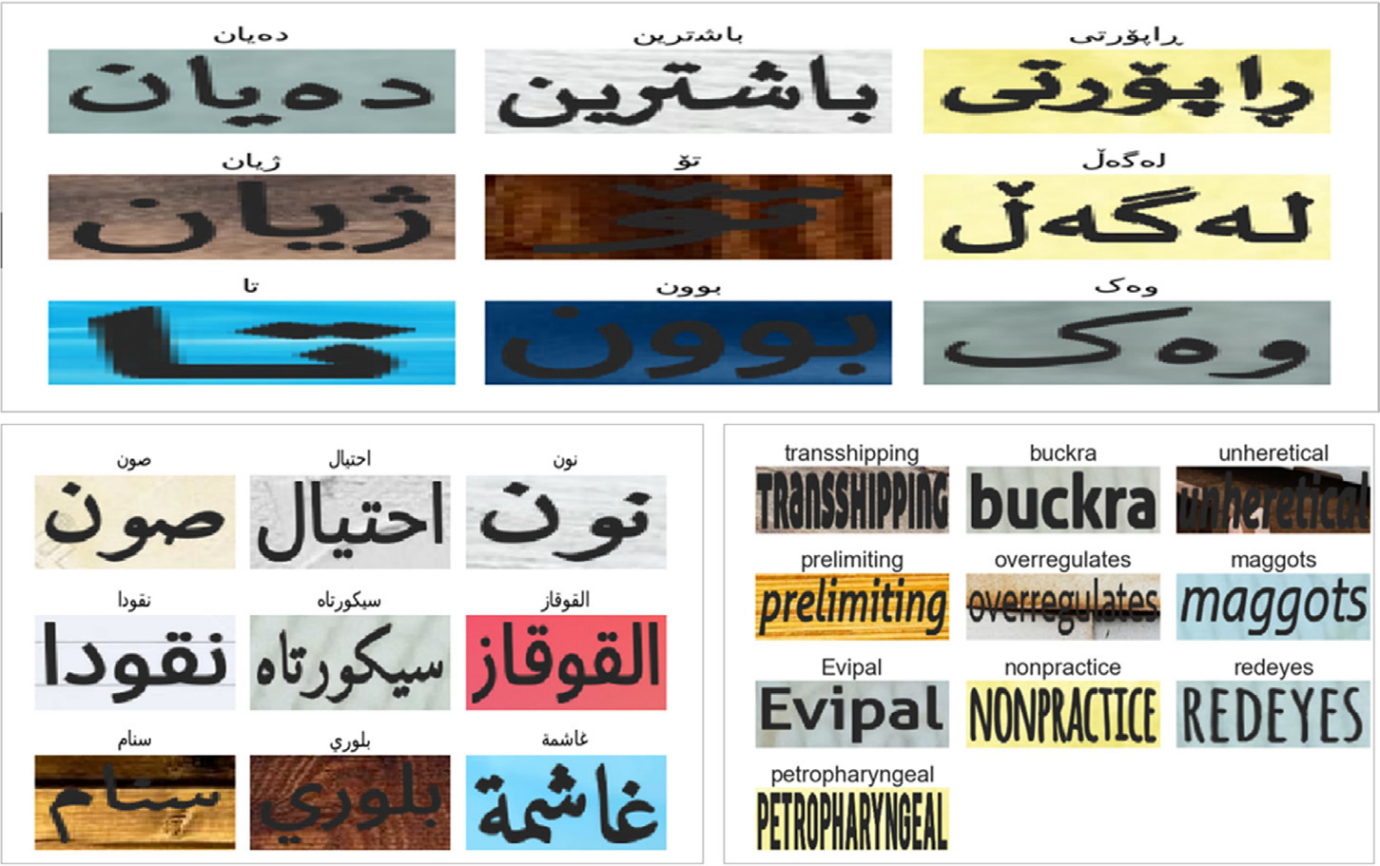
Samples of synthetic text images generated for Kurdish (top), Arabic (left), and English (right) illustrate diversity.

Fig. 12 illustrates examples of synthetically generated text images for Kurdish (top), Arabic (left), and English (right). These samples are designed to replicate real-world variations in font styles, text distortions, and environmental conditions, thereby enhancing the ability of models to generalize across diverse text appearances. The synthetic text generation process was carried out using PaddleOCR [21], incorporating a variety of fonts, text sizes, orientations, and background complexities to closely simulate real-world scenarios. Furthermore, different styles of Kurdish text were generated to represent both the Sorani and Badini dialects, ensuring that the dataset comprehensively captures the variations inherent in the Kurdish script.

The synthetic dataset helps overcome data scarcity in Kurdish Scene Text Recognition (KSTR) by generating high-quality training samples that complement real-world images. The variations in font styles, distortions and text orientations improve the robustness of text recognition models trained on this dataset.

**Fig. 13:**
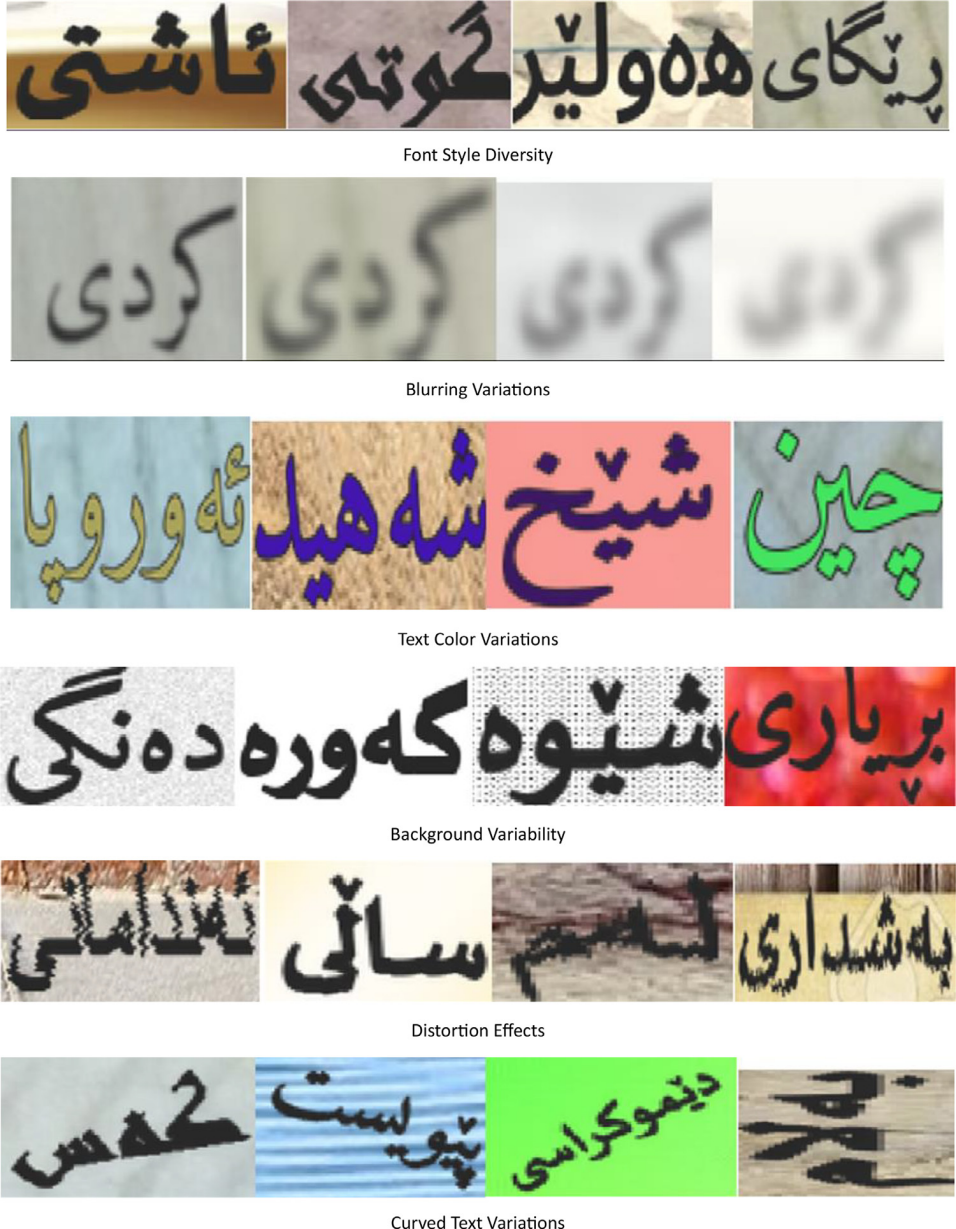
Samples of augmented synthetic text images simulating real-world challenges.

Some synthetic text images were augmented to better reflect real-world conditions, ensuring that models trained on KSTRV1 can generalise to diverse environments. These augmentations simulate real-world distortions, making the dataset more robust for Scene Text Recognition (STR) models. Fig. 13 presents a selection of synthetically generated text images that simulate real-world text recognition challenges.

### 4.6 Statistics

This dataset comprises natural scene images captured in the Kurdistan Region of Iraq, featuring text in Kurdish script, alongside English and Arabic scripts. The text appears in various combinations, including Kurdish only, Kurdish with Arabic, Kurdish with English, and Kurdish with both Arabic and English. Additionally, Kurdish text is represented in two dialects: Sorani and Badini. Table 4 presents the distribution of images in the dataset, categorized by Kurdish-only text and Kurdish text appearing alongside Arabic and English.

**Table 4:** Number of samples containing kurdish text alone and in combination with Arabic and English.

Image Container	Number of Sample
Kurdish	409
Kurdish With English	873
Kurdish With Arabic	423
Kurdish With English and Arabic	284

Table 5 presents a comparative analysis of the number of word instances in the dataset for each language. The left table displays the number of labelled text images across Kurdish, Arabic, and English, while the right table presents the word count in the synthetic data for each language. It is noteworthy that the numbers on the nature images can appear in two styles: English (0–9) and Hindi (٩-٠). Numbers written in Hindi are calculated based on their context, which may correspond to Arabic or Kurdish since both languages use Hindi digits, while English-style numbers are calculated across all three languages due to English numeric being common in three languages.

**Table 5:** Left number of nature scent text instances words and right number of synthetic text instance images.

Table 6 presents the top 10 most frequently annotated words across Kurdish, Arabic, and English. From this list, it is evident that the most common word in Kurdish is “و”, in Arabic is “و”, and in English is “&”.\

**Table 6:** Most frequency words in three languages.

## Limitations

Not applicable’

## Ethics Statement

The authors confirm that they have read and follow the ethical requirements for publication in *Data in Brief* and that the current work does not involve human subjects, animal experiments, or any data collected from social media platforms.

## CRediT authorship contribution statement

**Sardar Omar Salih:** Data curation, Methodology, Software. **Karwan Jacksi:** Supervision, Conceptualization, Writing – original draft.

## Data Availability

ZenodoKSTRV1 (Kurdish STR) Version 1 (Original data).
